# Charting the Neurotropism of Human Parvovirus B19 and Encephalitis: A Systematic Review of Existing Literature of 25 Years

**DOI:** 10.7759/cureus.89551

**Published:** 2025-08-07

**Authors:** Arham Amir Khawaja, Haseeb Mehmood Qadri, Momin Bashir, Ali Gohar, Muhammad Rafay Rafique, Haseeb Ahmad, Nawal Habib, Asma Kiran, Shoaib Khaliq, Asif Bashir

**Affiliations:** 1 General Surgery and Surgical Oncology, Shaikh Zayed Medical Complex, Lahore, PAK; 2 Neurological Surgery, Punjab Institute of Neurosciences, Lahore, PAK; 3 Psychology, Neuroscience, and Microbiology, New York University, New York, USA; 4 Pulmonology, Lahore General Hospital, Lahore, PAK; 5 Internal Medicine, Nawaz Sharif Medical College, Gujrat, PAK; 6 Internal Medicine, Allama Iqbal Medical College, Lahore, PAK; 7 General Surgery, Allama Iqbal Medical College, Lahore, PAK

**Keywords:** acyclovir, ataxia, central nervous system, encephalitis, immunocompromised, immunoglobulins, parvovirus b19, polymerase chain reaction

## Abstract

Parvovirus B19 (PVB19) is an infrequent, serious, yet treatable cause of infection in immunocompromised hosts. Neurological manifestations of PVB19 are encephalitis, encephalopathy, meningitis, cerebellar ataxia, transverse myelitis, stroke, and peripheral neuropathy. The objective is to identify the exact clinical and diagnostic features specific to parvovirus B19 encephalitis for the isolation and management of the pathology. This systematic review was conducted in September 2024 and included all the reported cases published between the years 2000 and 2024, following Preferred Reporting Items for Systematic Reviews and Meta-Analyses (PRISMA) guidelines. An extensive literature search was performed from PubMed Central and Google Scholar. The literature search was conducted using a set of predefined keywords and Boolean operators (AND/OR). All case reports, case series, and original articles containing complete and confirmed cases of human parvovirus B19 infection and presenting with features of encephalitis were selected. After detailed scrutiny, 36 articles were finally used in the review.

The mean age of the pediatric and adult patients was 5.78 ± 4.38 and 39.07 ± 19.4 years, respectively. About 59.7% (49) were men, 40.24% (33) were women, and 44.23% (46) did not present with any comorbidities. Most patients (29.03%) presented with altered sensorium. Fever was present in 19.35% (30) of the patients, and 14.19% (22) developed seizures. Around 47.12% (49) had no significant findings on examination. The majority of the patients had no co-infection with other viruses, which were about 88.89% (80) of cases. Polymerase chain reaction (PCR) tested positive in 36.89% (45 cases) of cerebrospinal fluid (CSF) samples. The majority of cases, 66.67% (16 patients), showed normal findings on computed tomography (CT) scans. The magnetic resonance imaging (MRI) findings in 22% (11) of the patients showed hyperintensity on diffusion-weighted imaging (DWI). Hyperintensity on T2-weighted imaging was seen in 14% (seven) of the patients. The parietal lobe was the most common site involved in five patients (11.90%). Acyclovir and intravenous immunoglobulin (IVIG) constituted the most common medications utilized, 13.01% each. Improvement was seen in 80.72% (67) after treatment.

In light of recent publications, the shift in the trend of parvovirus B19 toward the central nervous system (CNS) is alarming. Despite limited information, the site of brain parenchymal involvement (parietal lobe, cerebellar hemispheres, corpus callosum, frontal lobe, and ventricles) and specific radiological findings such as hyperintensity on DWI and T2-weighted images, along with CSF PCR positivity and neurological symptoms, can point toward parvovirus encephalitis.

## Introduction and background

Parvovirus B19 (PVB19) is a small non-enveloped single-stranded linear DNA virus [1]. It has three genotypes infecting humans only and belongs to the family of Parvoviridae [1]. It has three routes of transmission: respiratory droplets, transfusion of tissue and organs, and from mother to fetus [2]. PVB19 frequently affects immunocompromised individuals across all age groups but can also cause disease in healthy adults and the pediatric population below 15 years of age [3]. The clinical manifestations of PVB19 infection include erythema infectiosum, arthropathy, hydrops fetalis, and aplastic crisis [2,4]. Congenital immunodeficiencies, such as X-linked hyper immunoglobulin M (IgM) syndrome and Nezelof's syndrome, are linked with PVB19 [5]. Serology and polymerase chain reaction (PCR) methods are used for the detection of PVB19, but bone marrow biopsy is the gold standard for the detection of the virus [2].

Neurological manifestations of PVB19 are encephalitis, encephalopathy, meningitis, cerebellar ataxia, transverse myelitis, stroke, and peripheral neuropathy. Encephalitis and encephalopathy are the most common manifestations, representing 38.8% of neurological cases present in the current literature [6]. Patients with PVB19 encephalitis can present with fever, rash, seizures, altered sensorium, and focal neurological deficit as the most common presentations [6]. Patients infected with PVB19 can also present with an autoimmune pathology [7]. The criteria for the diagnosis of parvovirus encephalitis involve a constellation of clinical and laboratory findings, such as a characteristic rash of erythema infectiosum, with or without central nervous system (CNS) disease, with the detection of B19 DNA and anti-B19 antibodies in cerebrospinal fluid (CSF) [8]. The detection of anti-B19 antibodies and B19 DNA in serum and CSF can also be used as a diagnostic tool [4,9]. Computed tomography (CT) and magnetic resonance imaging (MRI) of the brain are used as radiological diagnostic tools for PVB19 [6]. There are no exact diagnostic criteria mentioned in the literature to differentiate PVB19 encephalitis from other neurotrophic viruses. No recent study was conducted just on typical and atypical manifestations of PVB19 encephalitis. The objective of our study was to bridge the literature gap by identifying the characteristic clinical findings and diagnostic markers specific to PVB19, thereby facilitating timely recognition, accurate diagnosis, and subsequently effective management protocol. In light of recent publications and epidemics, we aim to amplify and review the findings in recently published cases to broaden the horizon.

## Review

Methodology

**Figure 1:**
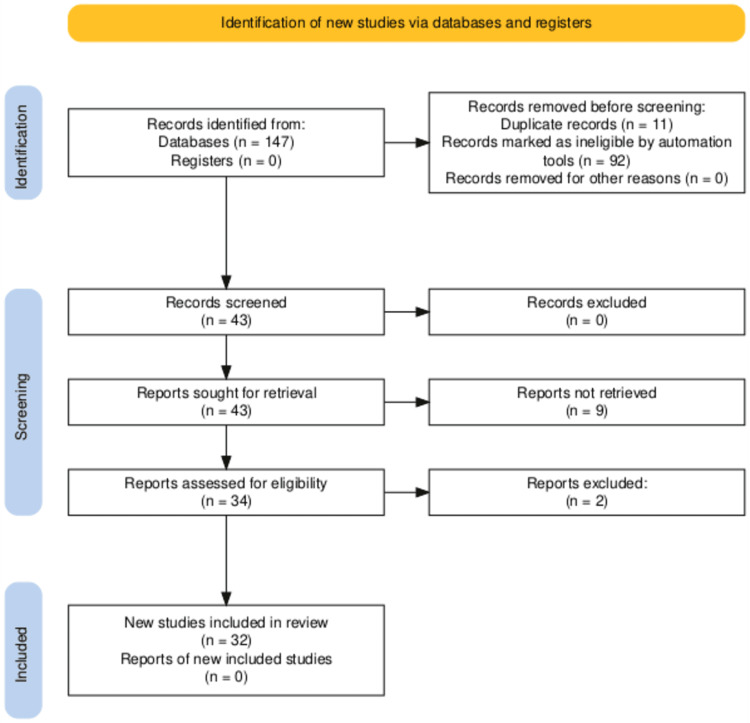
Preferred Reporting Items for Systematic Reviews and Meta-Analyses (PRISMA) flowchart for search strategy and the quality assessment of included studies. Note: Initial search of the Google Scholar database using predefined keywords yielded approximately 9,670 results, which were reduced to just 25 studies based on relevance. Similarly, a search on PubMed identified a total of 122 articles.

This systematic review was conducted to encompass the clinical manifestations, diagnostic features, and management for patients developing encephalitis in confirmed parvovirus B19 infection. The review was conducted via Preferred Reporting Items for Systematic Reviews and Meta-Analyses (PRISMA) guidelines (Figure 1).

Search Strategy

This review encompasses all reports published between 1 January 2000 and 1 August 2024, incorporating all old and recently published cases of confirmed cases of parvovirus B19 encephalitis. An extensive literature search was performed from PubMed Central and Google Scholar. The search was conducted using predefined keyword combinations and Boolean operators (AND/OR). The keywords used are stated as follows: "Human Parvovirus B19 encephalitis", "Human Parvovirus B19 meningoencephalitis", "Human Parvovirus B19 myalgic encephalitis", "Parvovirus B19 infection", "Parvovirus B19" AND "Brain", "Parvovirus B19" AND "CNS", "Parvovirus B19" AND "Central Nervous System", "Human Parvovirus B19 infection", "Human Parvovirus B19 Fever", "Human Parvovirus B19" AND "viremia", "Congenital Parvovirus Infection", and "Congenital Parvovirus B19". The articles were isolated according to predefined inclusion and exclusion criteria.

Inclusion Criteria

All case reports, case series, and original articles containing complete and confirmed cases of human parvovirus B19 infection and presenting with features of encephalitis and all articles that adhered to the English language and were open access with complete information were selected in our study.

Exclusion Criteria

Editorial articles, pictorial essays, letters to editors, and short communications and articles that were in languages other than English and non-open access or contained titles/abstracts only were excluded. Studies involving animals, cadavers, and infections by other parvovirus subtypes were also excluded.

Data Collection and Analysis

Data was collected by five independent data collectors, HMQ, AAK, HA, AK, and NH, who extensively searched the existing literature within the defined time frame. Data entered by respective collectors was cross-checked by HMQ to avoid selection bias. After scrutinizing the articles, the data was extracted and entered into pre-made tables in Microsoft Word 365 (Microsoft Corp., Redmond, WA). The tables consisted of information on demographic details (age and gender), details of publication, presenting features, examination findings, laboratory and radiological investigation, treatment, and management protocol. Finally, data on preexisting conditions, associated non-neurological findings, and genetic and autoimmune predilection was also added. Data entered by respective collectors was cross-checked to avoid selection bias. All extracted data was imported into the Statistical Package for Social Sciences software (IBM SPSS Statistics for Windows, version 24, IBM Corp., Armonk, NY) for statistical analysis. Descriptive statistics were used to summarize the data. Tables were generated to illustrate the findings.

Data Stratification

A search of the Google Scholar database using predefined keywords yielded approximately 9,670 results, which were reduced to just 25 studies based on relevance. Similarly, a search on PubMed identified a total of 122 articles. A total of 147 articles were identified by keyword search on PubMed and Google Scholar. After the initial screening, 11 articles were removed due to duplication, and a further 92 articles were removed as they were deemed ineligible, as they discussed non-neurological manifestations. The quality of the included studies was evaluated using the Joanna Briggs Institute (JBI) Critical Appraisal Checklist. The assessment criteria included study design, sample representativeness, case definition, the measurement of outcomes, and statistical analysis. Studies were rated according to predetermined criteria, with quality scores assigned accordingly. All 43 articles passed the JBI assessment criteria. Nine articles were excluded as they had missing information or were not retrievable. Two articles were excluded as they discussed another parvovirus subtype. A total of 32 articles were selected for the final review (Figure 1).

Results

A total of 32 articles were isolated, containing data on 88 patients. Most of the publications were in the year 2008, accounting for about 15.63% of the total. In 2001, 12.50% of the articles were published. Articles published in 2017, 2016, and 2006 accounted for about 9.38% each.

Most of the publications were from Japan (18.75%), followed by Italy (12.50%). China, Brazil, the United States, France, Turkey, Australia, and the United Kingdom each contributed 6.25% (Table 1).

**Table 1:** Geographic distribution of articles included in the study. **Portugal, India, South Korea, Tunisia, the Netherlands, Germany, the West Indies, and Tucson.

Country of publication	Frequency (n)	Percentage (n/N)
Japan	6	18.75%
Italy	4	12.50%
China	2	6.25%
Brazil	2	6.25%
United States	2	6.25%
France	2	6.25%
Turkey	2	6.25%
Australia	2	6.25%
United Kingdom	2	6.25%
Miscellaneous**	8	25.04%
Total (N)	32	100%

The mean age of the pediatric patients was 5.78 ± 4.38 years, and that of adult patients was 39.07 ± 19.4 years (Table 2). The percentage of pediatric patients was 56.79% (46), and adult patients were 43.21% (35) (Table 3).

**Table 2:** Mean age and standard deviation of adult and pediatric patients.

Age group	Mean (years)	Standard deviation (years)
Pediatric	5.78	4.38
Adult	39.07	19.4

**Table 3:** Age distribution and percentage occurrence of reported cases.

Age group	Frequency (n)	Percentage (n/N)
Pediatric	46	56.79%
Adult	35	43.21%
Total (N)	81	100%

The percentage of men was 59.7% (49), and the percentage of women was 40.24% (33) (Table 4).

**Table 4:** Gender distribution with percentage occurrence of the reported cases.

Gender	Frequency (n)	Percentage (n/N)
Male	49	59.75%
Female	33	40.24%
Total (N)	82	100%

The majority of the patients did not present with any co-morbidities, corresponding to 44.23% (46). About 9.62% (10) were diagnosed with sickle cell disease, and 7.69% (8) had hepatitis/liver failure/cirrhosis (Table 5).

**Table 5:** Co-morbid conditions reported with frequency and percentage occurrence. HIV, human immunodeficiency virus; DIC, disseminated intravascular coagulation

Co-morbid conditions	Frequency (n)	Percentage (n/N)
No co-morbidities/not mentioned	46	44.23%
Sickle cell disease	10	9.62%
Hepatitis/liver failure/cirrhosis	8	7.69%
Congenital heart disease	6	5.77%
Hereditary spherocytosis	4	3.85%
Cerebrovascular accidents	3	2.88%
Leukemia	3	2.88%
Renal failure	3	2.88%
Cockayne syndrome	2	1.92%
Necrotizing enterocolitis	2	1.92%
Osteopenia	2	1.92%
Turner syndrome	2	1.92%
DIC	2	1.92%
Mitral valve prolapse	1	0.96%
Sjögren syndrome	1	0.96%
Anti-synthetase syndrome	1	0.96%
Tuberculosis	1	0.96%
Hypertension	1	0.96%
Renal transplant	1	0.96%
HIV	1	0.96%
Herpes simplex infection	1	0.96%
Crigler-Najjar syndrome	1	0.96%
Polycystic kidney disease	1	0.96%
Hemophagocytic lymphohistiocytosis	1	0.96%
Total (N)	104	100%

Three patients had an infected close family member. One patient had an intrauterine mode of transmission of B19. The mode of transmission of the rest of the patients is not known.

The majority of the patients (29.03%) presented with altered sensorium, who were about 45 in number. Fever was exhibited in 19.35% (30) of patients, and 14.19% (22) developed seizures (Table 6).

**Table 6:** Clinical manifestations at the presentation of reported patients. GBS: Guillain-Barré syndrome

Clinical manifestations	Frequency (n)	Percentage (n/N)
Altered sensorium (confusion, disorientation, drowsiness, and loss of consciousness)	45	29.03%
Fever	30	19.35%
Seizures	22	14.19%
Headache	13	8.39%
Limb/respiratory muscle paralysis	13	8.39%
Behavioral issues/neuropsychiatric	8	5.16%
Eye symptoms (diplopia, pain, blurring, asymmetry, and ocular revulsion)	5	3.23%
Irritability/agitation	4	2.58%
GBS	3	1.94%
Delusions/hallucinations	3	1.94%
Slurred speech and dysarthria	3	1.94%
Lethargy/torpid	2	1.29%
Facial twitching	1	0.65%
Paresthesia	1	0.65%
Motor disorder	1	0.65%
Aphasia	1	0.65%
Total (N)	155	100%

Most of the patients (47.12%) had no significant findings on examination, corresponding to 49 cases. About 15.38% (16) of the patients showed the positive findings of meningeal irritation, and 3.85% (four) of the patients had ataxia/gait disturbances (Table 7).

**Table 7:** Physical examination of the reported patients. CN: cranial nerve

Examination findings	Frequency (n)	Percentage (n/N)
No findings/not mentioned	49	47.12%
Meningeal irritation (e.g., neck stiffness)	16	15.38%
Ataxia/gait disturbance	4	3.85%
Hyperreflexia	3	2.88%
Tremors	3	2.88%
Reduced visual acuity	2	1.92%
Dysmetria	2	1.92%
Sensory loss	2	1.92%
Expressive dysphasia	2	1.92%
Rigidity (pyramidal and extrapyramidal)	2	1.92%
Tremors	1	0.96%
Cerebral ataxia	1	0.96%
Limb paresis	1	0.96%
Relative afferent pupillary defect	1	0.96%
Anisocoria	1	0.96%
Dilated pupil	1	0.96%
Constricted visual fields	1	0.96%
Romberg sign	1	0.96%
Hypertonia	1	0.96%
Strabismus	1	0.96%
Nystagmus	1	0.96%
Nuchal rigidity	1	0.96%
Hypotonia	1	0.96%
Dysarthria	1	0.96%
Hyporeflexia	1	0.96%
Apraxia	1	0.96%
Facial paresthesia	1	0.96%
Paralysis of CN VII	1	0.96%
Urinary incontinence	1	0.96%
Total (N)	104	100%

The majority of the patients (30.38%) had no non-neurological symptoms, which were about 37 cases. Hepatitis/liver failure was positive in 9.17% (11) of the patients, and 8.33% (10) of the patients had findings consistent with pulmonary infections (Table 8).

**Table 8:** Concomitant non-neurological symptoms of the reported patients.

Associated non-neurological symptoms	Frequency (n)	Percentage (n/N)
Not mentioned	37	30.83%
Hepatomegaly/hepatitis/liver failure	11	9.17%
Respiratory symptoms/pulmonary infection	10	8.33%
Rash	8	6.67%
Pancytopenia/aplastic crisis	7	5.83%
Arthralgia/arthritis	4	3.33%
Lymphadenitis/lymphadenopathy	4	3.33%
Yellowing of the bulbar conjunctiva/jaundice	3	2.50%
General fatigue	3	2.50%
Splenomegaly	3	2.50%
Thrombocytopenia	3	2.50%
Hemolytic anemia	2	1.67%
Cyanosis	2	1.67%
Sore throat	2	1.67%
Acute renal failure	2	1.67%
Disseminated intravascular coagulation	2	1.67%
Gastroenteritis/abdominal pain	2	1.67%
Ascites	2	1.67%
Proteinuria	2	1.67%
Hypertension	2	1.67%
Acute chest syndrome	2	1.67%
Epigastric pain	1	0.83%
Parotid swelling	1	0.83%
Conjunctivitis	1	0.83%
Hemophagocytic lymphohistiocytosis	1	0.83%
Cockayne syndrome	1	0.83%
Necrotizing enterocolitis	1	0.83%
Myalgia	1	0.83%
Total (N)	120	100%

Most patients (88.89%, 80 cases) had no co-infection with other viruses, while 5.56% (five cases) tested positive for herpes simplex virus (HSV) (Table 9).

**Table 9:** Percentage of patients under study having co-infection with other viruses. HSV, herpes simplex virus; HIV, human immunodeficiency virus; HCV, hepatitis C virus; VZV, varicella-zoster Virus; CMV, cytomegalovirus

Co-infection with other viruses	Frequency (n)	Percentage (n/N)
No co-infection	80	88.89%
HSV	5	5.56%
HIV	1	1.11%
HCV	1	1.11%
VZV	1	1.11%
Enterovirus	1	1.11%
CMV	1	1.11%
Total (N)	90	100%

The majority of the patients (36.89%) had CSF PCR positivity, about 45 cases. Normal glucose levels were seen in 13.93% (17) of the patients. The data for 12.30% of the patients was not given. Elevated protein levels in CSF were observed in 9.84% (12) (Table 10).

**Table 10:** Cerebrospinal fluid and serum findings in the reported patients. CSF, cerebrospinal fluid; PCR, polymerase chain reaction

CSF and serum findings	Frequency (n)	Percentage (n/N)
CSF PCR-positive	45	36.89%
Normal glucose	17	13.93%
Not mentioned	15	12.30%
Elevated protein	12	9.84%
High WBCs	10	8.20%
Serum PCR-positive	8	6.56%
Normal protein	7	5.74%
Pleocytosis	5	4.10%
Normal WBCs	3	2.46%
Elevated glucose	1	0.82%
Low glucose	1	0.82%
Low protein	1	0.82%
Xanthochromic appearance	1	0.82%
Lymphocyte predominant	1	0.82%
Total (N)	122	100%

The majority of the patients (66.67%) had normal findings on the CT scan, found in 16 cases. About 16.67% (four) had an undefined lesion on CT imaging. Edema, enhancement, enlargement, and dilation were seen in 4.17% (one) of the patients each.

MRI findings in 22% (11) of the patients showed hyperintensity on diffusion-weighted imaging (DWI). About 14% (seven) of the patients showed hyperintensity on T2, and 10% (five) of the patients showed hyperintensity on fluid-attenuated inversion recovery (FLAIR) and features consistent with inflammation, respectively (Table 11).

**Table 11:** Summary of computed tomography (CT) and magnetic resonance imaging (MRI) findings. DWI, diffusion-weighted imaging; FLAIR, fluid-attenuated inversion recovery

Radiology findings	Frequency (n)	Percentage (n/N)
CT findings		
Normal findings	16	66.67%
Undefined lesion	4	16.67%
Edema	1	4.17%
Enhancement	1	4.17%
Enlargement	1	4.17%
Dilation	1	4.17%
Total (N)	24	100%
MRI findings		
Hyperintensity on DWI	11	22.00%
Hyperintensity on T2	7	14.00%
Hyperintensity on FLAIR	5	10.00%
Inflammation	5	10.00%
Normal findings	5	10.00%
Enhancement	4	8.00%
Dilation	3	6.00%
Enlargement	3	6.00%
Expansion	1	2.00%
Patchy, longer signals on T2	1	2.00%
Polymicrogyria	1	2.00%
Heterotropia	1	2.00%
Cortical atrophy	1	2.00%
Gliosis	1	2.00%
Wide spaces	1	2.00%
Total (N)	50	100%

The most common site of the brain involved was the parietal lobe (11.90%), which was found in five cases. Cerebellar hemispheres, the corpus callosum, the frontal lobe, and all ventricles were involved in 9.52% (four) of cases each (Table 12). 

**Table 12:** Sites of the brain involved with frequency and percentage occurrence.

Sites of the brain involved	Frequency (n)	Percentage (n/N)
Parietal lobe	5	11.90%
Cerebellar hemispheres	4	9.52%
Corpus callosum	4	9.52%
Frontal lobe	4	9.52%
All ventricles	4	9.52%
Temporal lobe	3	7.14%
Basal ganglia	2	4.76%
Occipital lobe	2	4.76%
Periventricular white matter	2	4.76%
Choroid plexus	2	4.76%
Thalamus	1	2.38%
Midbrain	1	2.38%
Leptomeningeal	1	2.38%
Centrum semiovale	1	2.38%
Lateral ventricle	1	2.38%
Infratentorial region	1	2.38%
Frontal gyrus	1	2.38%
Post central gyrus	1	2.38%
Caudate nucleus	1	2.38%
Lenticular nucleus	1	2.38%
Total	42	100%

Improvement was seen in most patients (80.72%) after treatment. About 8.43% (seven) of the patients deteriorated after treatment, while 8.43% (seven) of the patients' conditions remained static (Table 13).

**Table 13:** Post-treatment sequelae of patients under study.

Post-treatment sequelae	Frequency (n)	Percentage (n/N)
Improved	67	80.72%
Deteriorated	7	8.43%
Static	7	8.43%
Not mentioned	2	2.41%
Total (N)	83	100%

All 71 patients underwent medical management. The treatment modality of the 12 patients is not mentioned.

A total of 85.88% of the patients (73 cases) survived, while 10.59% (nine cases) resulted in death. Data was not given for three patients (3.53%) (Table 14).

**Table 14:** Final outcome of all reported patients.

Outcomes	Frequency (n)	Percentage (n/N)
Alive	73	85.88%
Dead	9	10.59%
Not mentioned	3	3.53%
Total (N)	85	100%

No genetic element was isolated predisposing to parvovirus B19 infection and encephalitis. Autoantibodies were present in only a few of the cases (Table 15).

**Table 15:** Autoantibodies associated with parvovirus B19 infection. GABA, gamma-aminobutyric acid; IgG, immunoglobulin G; ANA, antinuclear antibody; GD1b, ganglioside D1b

Autoantibodies	Frequency (n)	Percentage (n/N)
Not mentioned or tested	78	89.66%
Negative	4	4.60%
Autoantibodies targeting the GABA receptor	1	1.15%
Anti-GD1b IgG	1	1.15%
ANA	1	1.15%
Lupus anticoagulant	1	1.15%
Anti-smooth muscle	1	1.15%
Total (N)	87	100%

Most of the treatment modalities were not mentioned. However, acyclovir and intravenous immunoglobulin (IVIG) constituted the most common medications utilized, each at 13.01% (19). Similarly, glucocorticoids were the second most popular medications used (10.96%), in about 19 cases (Table 16). The follow-up of 25 patients is mentioned. The mean follow-up of patients was 55.71 ± 131.07 months.

**Table 16:** Treatment protocol used in different patients. IVIG: intravenous immunoglobulin

Medications	Frequency (n)	Percentage (n/N)
Not mentioned	44	30.14%
Acyclovir	19	13.01%
IVIG	19	13.01%
Glucocorticoid (methylprednisolone and prednisone)	16	10.96%
Antibiotics	15	10.27%
Ganciclovir	12	8.23%
Anti-seizure (phenobarbital, carbamazepine, topiramate, midazolam, etc.)	12	8.23%
Immunosuppressant (cyclosporine, azathioprine, etc.)	6	4.11%
Penciclovir	1	0.68%
Thrombomodulin	1	0.68%
Mannitol	1	0.68%
Total (N)	146	100%

The details of the studies that were included in the final review are listed below (Table 17).

**Table 17:** Details of studies included with year and country of publication.

Study	Study title	Year of publication	Country of publication
Barah et al. [9]	Association of human parvovirus B19 infection with acute meningoencephalitis	2001	United Kingdom
Valle et al. [10]	GABAA receptor encephalitis associated with human parvovirus B19 virus infection	2021	Brazil
Guo et al. [11]	Human parvovirus B19 infection in hospitalized patients suspected of infection with pathogenic microorganism	2022	China
Cao and Zhu [12]	Acute viral encephalitis associated with human parvovirus B19 infection: unexpectedly diagnosed by metagenomic next-generation sequencing	2020	China
Shinagawa et al. [13]	Human parvovirus B19-associated encephalopathy with hereditary spherocytosis	2019	Japan
Sequeira et al. [14]	Parvovirus B19 infection associated with hemolytic anemia and cranial polyneuropathy	2017	Portugal
Kumar et al. [15]	Epidemiological profile of acute viral encephalitis	2017	India
Jun et al. [16]	Clinical manifestations and treatment outcomes of parvovirus B19 encephalitis in immunocompetent adults	2017	South Korea
Takasawa et al. [17]	Steroid-responsive Status Epilepticus caused by human parvovirus B19 encephalitis	2016	Japan
Palermo et al. [18]	Focal epilepsy as a long term sequela of Parvovirus B19 encephalitis	2016	Italy
Bouafsoun et al. [19]	Prevalence of human parvovirus B19 infection during febrile rashes in children in northern Tunisia	2016	Tunisia
Suzuki et al. [20]	Clinically mild encephalitis/encephalopathy with a reversible splenial lesion caused by human parvovirus B19 infection: a case of two brothers with hereditary spherocytosis	2014	Japan
Shroff et al. [21]	An unusual cause of cerebellar ataxia in an immunocompromised elderly patient	2014	United States
Uchida et al. [22]	Acute cerebellitis and concurrent encephalitis associated with parvovirus B19 Infection	2012	Japan
Meyer et al. [23]	Parvovirus B19 encephalitis in children	2011	France
Pistorius et al. [24]	Disturbance of cerebral neuronal migration following congenital parvovirus B19 infection	2008	Netherlands
Oshima et al. [25]	Acute encephalopathy with human parvovirus B19 infection in hereditary spherocytosis	2008	Japan
Greco et al. [26]	Severe ataxia as a complication of human parvovirus B19 acute encephalitis in a child	2008	Italy
Coskun et al. [27]	Meningoencephalitis associated with human parvovirus B19	2008	Turkey
Bonvicini et al. [28]	Meningoencephalitis with persistent parvovirus B19 Infection in an apparently healthy woman	2008	Italy
Tonnellier et al. [29]	A possible parvovirus B19 encephalitis in an immunocompetent adult patient	2007	France
Hammond et al. [30]	Parvovirus B19 infection of brain: possible role of gender in determining mental illness and autoimmune thyroid disorders	2007	United States
Steinfort and Dixon [31]	Parvovirus encephalitis and pneumonia in an immunocompetent adult	2006	Australia
Laurenz et al. [32]	Severe parvovirus B19 encephalitis after renal transplantation	2006	Germany
Erol et al. [33]	Refractory status epilepticus owing to human parvovirus B19 encephalitis in a child	2006	Turkey
Nolan et al. [34]	Parvovirus B19 encephalitis presenting as immune restoration disease after highly active antiretroviral therapy for human immunodeficiency virus infection	2003	Australia
Guidi et al. [35]	Case of stroke in a 7-year-old male after parvovirus B19 infection	2003	Italy
Yazawa et al. [36]	Case report of meningoencephalitis during a concomitant mumps and parvovirus B19 infection	2002	Japan
Kerr et al. [37]	Evidence for the role of demyelination, HLA-DR alleles, and cytokines in the pathogenesis of parvovirus B19 meningoencephalitis and its sequelae	2002	United Kingdom
Wierenga et al. [38]	Cerebrovascular complications and parvovirus infection in homozygous sickle cell disease	2001	West Indies
Skaff and Labiner [39]	Status epilepticus due to human parvovirus B19 encephalitis in an immunocompetent adult	2001	Tucson
Pereira et al. [40]	Two family members with a syndrome of headache and rash caused by human parvovirus B19	2001	Brazil

Discussion

Among the studies included in this systematic review, the most frequently represented year of publication was 2008, accounting for five studies, followed by 2001 with four studies. The years 2006, 2016, and 2017 each contributed three studies to the review. This distribution indicates a consistent and sustained research interest in parvovirus B19 over the past two decades. Considering that the virus was first identified in 1975, the noticeable increase in publications during the early 2000s is not unexpected, reflecting a period of intensified investigation, likely driven by advancements in diagnostic techniques, increased awareness of the virus's clinical implications, and a growing recognition of its relevance in various medical contexts [5].

In terms of geographic distribution, Japan emerged as the leading contributor, with six studies (18.75%), followed by Italy with four studies (12.50%). Additionally, China, Brazil, the United States, France, Turkey, Australia, and the United Kingdom each contributed two studies (6.25%). This international spread of research efforts highlights the global concern surrounding parvovirus B19 and underscores its significance across diverse healthcare systems. The distribution of studies aligns well with broader epidemiological trends and reflects the active involvement of both Western and Eastern countries in furthering the scientific understanding of this virus [6].

The mean ages of pediatric and adult patients were 5.78 ± 4.38 and 39.07 ± 19.4 years, respectively, both of which are lower than the mean ages reported in comparable studies [4,6]. Pediatric patients represented a slight majority of the cohort, comprising 56.79%, while adult patients accounted for 43.21%. This distribution is consistent with the established epidemiology of parvovirus B19, which tends to infect children more frequently. However, seroprevalence studies indicate higher antibody levels in adults, particularly among the elderly population [41,42]. This elevated seroprevalence in older age groups is likely attributable to the cumulative effect of subclinical infections over time, which results in the gradual development of immunity.

With regard to gender distribution, 59.75% of the patients were men, compared to 40.24% who were women. Although this study shows a modest male predominance, the literature reveals variability in sex distribution, with some studies reporting a higher incidence in men and others showing more balanced or even female-skewed data [4,6]. These differences may be influenced by factors such as population demographics, study design, and regional variation in exposure risk.

A notable proportion of patients in this study did not present with any comorbidities. Among those who did, the most prevalent conditions were sickle cell disease and acute and chronic hepatitis, affecting 9.62% and 7.69% of the cohort, respectively. These findings are consistent with existing literature, which identifies a higher frequency of parvovirus B19 infections in individuals with underlying hematologic and hepatic conditions [43,44].

The association between parvovirus B19 and sickle cell disease is particularly well-documented. This virus specifically targets erythroid progenitor cells in the bone marrow, resulting in a temporary suppression of red blood cell production. In patients with sickle cell anemia, who already experience chronic hemolysis, this suppression can precipitate an aplastic crisis. Furthermore, the frequent need for blood transfusions in these patients may increase their exposure to the virus. Similarly, individuals with hepatitis or liver dysfunction may be more vulnerable to parvovirus B19 due to compromised immune responses, which can reduce their ability to effectively clear viral infections [13,14,20]. These interactions underscore the importance of monitoring at-risk populations for potential parvovirus B19 co-infections and their clinical consequences.

The clinical manifestations of parvovirus B19 infections are notably varied, with the most commonly reported symptoms in this study being altered sensorium, followed by fever and seizures. These findings align with the symptomatology described in other studies, which also highlight neurological involvement as a prominent feature of parvovirus B19 infections [5,6]. On clinical examination, the majority of patients had no significant abnormalities. However, 15.38% exhibited signs of meningeal irritation, and 3.85% presented with ataxia or gait disturbances. The predominance of patients with no significant findings is likely reflective of the fact that parvovirus B19 infections are often asymptomatic, especially in individuals with intact immune systems [41,42]. The presence of ataxia or gait disturbances in a subset of patients is consistent with the potential for parvovirus B19 to cause neurological complications, such as cerebellar involvement or peripheral neuropathies, which have been observed in more severe cases [41,42]. These findings emphasize the importance of clinical vigilance, particularly in pediatric populations, where parvovirus B19 can sometimes lead to more pronounced neurological sequelae.

In terms of non-neurological symptoms, the majority of patients in this cohort reported no clinically significant manifestations, with 9.17% presenting with hepatomegaly, hepatitis, or liver failure and 8.33% exhibiting respiratory symptoms or pulmonary infections. These observations are consistent with prior reports and may be attributed to similar pathophysiological mechanisms underlying these conditions in the context of parvovirus B19 infection [42]. Furthermore, 88.89% of the patients did not show any evidence of co-infection with other viral pathogens. However, 5.56% tested positive for herpes simplex virus (HSV). The relatively low frequency of HSV co-infection observed in this study suggests that HSV is unlikely to play a significant role in the clinical course or outcome of parvovirus B19 infection, supporting the notion that its presence does not significantly alter the clinical manifestations in this population.

The majority of patients in this study exhibited normal CT findings, indicating no significant structural abnormalities. However, 16.67% of the patients presented with undefined lesions, which complicate the ability to establish a direct association between parvovirus B19 infection and these lesions. Notably, similar undefined lesions have been reported in previous studies, suggesting that such findings may not be uncommon in the context of parvovirus B19 infection [4,6]. A small proportion of patients demonstrated CT abnormalities, including edema, enhancement, or enlargement. MRI findings further revealed that 22.00% of the patients showed hyperintensities on DWI, 14.00% exhibited hyperintensities on T2-weighted imaging, and 10.00% each presented with hyperintensities on FLAIR imaging, inflammation, or normal results. The hyperintensities observed on T2 and FLAIR imaging are likely indicative of increased water content or edema, given that both sequences are sensitive to these changes. The presence of edema on CT could thus correspond with the hyperintensities seen on T2 and FLAIR MRI sequences. Additionally, the enlargement observed on MRI may be related to the lesions detected on CT; however, it is important to note that such enlargement is typically more characteristic of neoplastic processes or significant structural alterations, rather than benign lesions. The enhancement observed on MRI, suggesting contrast uptake, could be indicative of an inflammatory process, which might also explain the inflammation detected on MRI.

The parietal lobe was the most commonly involved site, affected in 11.90% of the patients, followed by the cerebellar hemispheres, corpus callosum, frontal lobe, and all ventricles, each of which was affected in 9.52% of patients. These findings suggest that parvovirus B19 may have broader neurological implications, potentially reflecting secondary complications or a more diffuse impact on the brain, rather than direct, primary effects confined to specific areas. The involvement of multiple brain regions, in conjunction with the CT and MRI findings observed in this cohort, points to a possible association with encephalitis, an inflammatory condition of the brain that has been previously linked to parvovirus B19, particularly in pediatric populations [6]. The diversity of symptoms observed in these patients, along with the documented imaging abnormalities and affected brain sites, further supports this potential association and aligns with the clinical manifestations of encephalitis as reported in the existing literature. Specifically, encephalitis is often characterized by diffuse brain involvement, including both cortical and subcortical regions, and is frequently accompanied by nonspecific findings such as edema, hyperintensities, and enlargement on imaging. Collectively, this data underscores the need for continued investigation into the mechanisms by which parvovirus B19 may contribute to encephalitis and other neurological complications.

Post-treatment, a significant majority of patients (80.72%) showed improvement in their condition, while only a small proportion experienced deterioration or remained unchanged, each group representing 8.43% of the total. The overall survival rate was 85.88%, indicating a relatively low mortality rate associated with parvovirus B19 infection, with 10.59% of the patients dying. These findings are consistent with data from other studies, although mortality rates may vary, particularly in the case of fetal infections [1,8]. The positive treatment outcomes suggest that the therapeutic interventions for parvovirus B19 infection are generally effective. The patients who did not survive either were likely immunocompromised or had significant comorbidities, which may have contributed to their complications and subsequent mortality. Additionally, 89.66% of the patients did not undergo autoantibody testing, and only 4.60% of those who were tested returned negative results. This suggests that no clear genetic predisposition to parvovirus B19 infection or associated encephalitis was identified in this cohort.

With the findings derived from a cohort of patients, we propose a flowchart that represents a stepwise approach to proceed in case of suspected parvovirus B19 infection presenting as encephalitis (see Appendices).

Limitations

Out of the 88 patients, a significant amount of data was missing across different categories. To highlight, data for 37 patients was not mentioned in the non-neurological symptomatology. Similarly, 49 patients were categorized as having "no findings," or their data was not mentioned in the examination findings, and 46 patients had missing data in the comorbidities. Additionally, the study focused exclusively on parvovirus B19, excluding other viral subtypes. The presence of viral co-infections might also lead to overlapping symptoms, complicating the interpretation of findings. There was no specific diagnostic marker identified that could specify the encephalitis caused by parvovirus B19. Finally, the study did not identify any genetic or autoimmune elements, limiting its applicability to those aspects.

## Conclusions

This review paves a path to a wider understanding of the complex pathophysiology of a single strain of parvovirus B19. While the brain and its mechanisms are an enigma in themselves, it is clear that we are just looking at the tip of an iceberg in our understanding of how viruses affect the brain. Currently, there are no recently predecided guidelines to differentiate between different viral encephalitides. However, we can derive a possible diagnosis of parvovirus B19 encephalitis by the site of brain parenchymal involvement (parietal lobe, cerebellar hemispheres, corpus callosum, frontal lobe, and ventricles) and specific radiological findings of hyperintensity on DWI and T2 and CSF PCR positivity coupled with neurological symptoms (altered sensorium, fever, and seizures). This review can, therefore, add an adjunct to diagnosis and possibly the early identification of such cases, especially in view of recent epidemics.

## Figures and Tables

**Figure 2:**
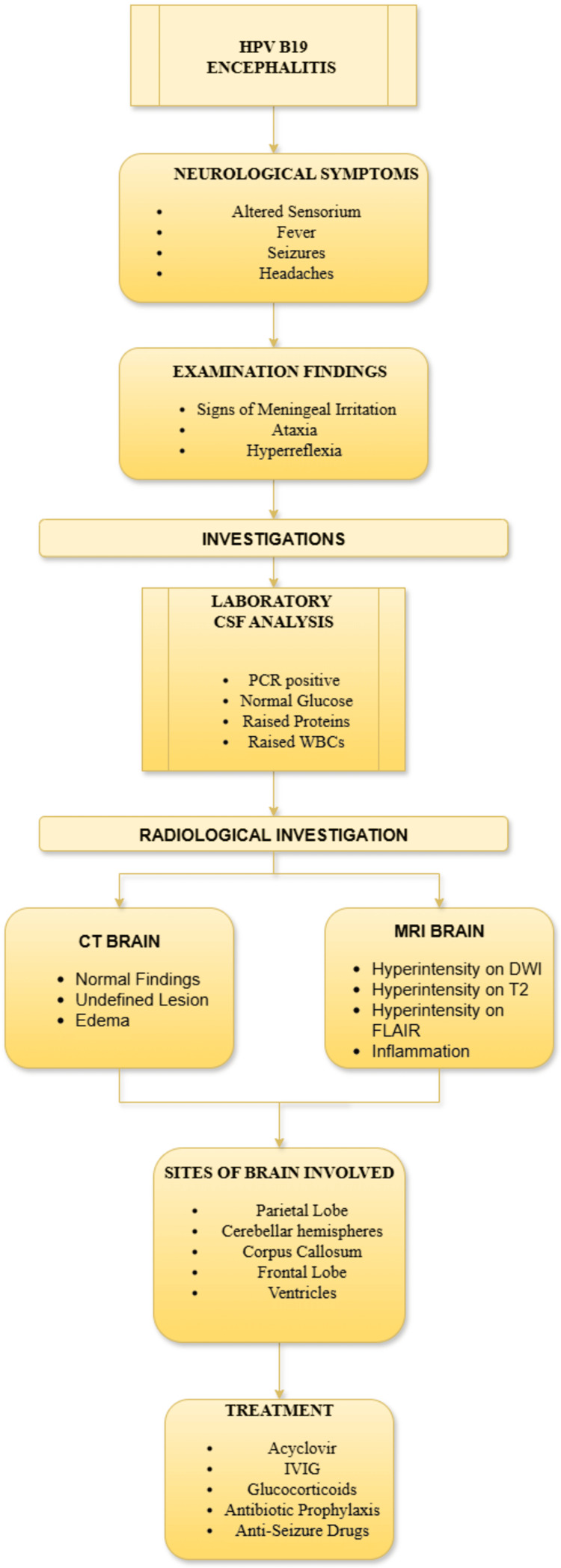
Author-proposed schematic diagram for the management of human parvovirus (HPV) B19 encephalitis. DWI, diffusion-weighted imaging; FLAIR, fluid-attenuated inversion recovery; PCR, polymerase chain reaction; CT, computed tomography; MRI, magnetic resonance imaging; IVIG, intravenous immunoglobulin; CSF, cerebrospinal fluid

## Appendices

Figure 2 shows a flowchart that represents a stepwise approach to proceed in case of suspected parvovirus B19 infection presenting as encephalitis.
